# Latent infection of myeloid progenitors by human cytomegalovirus protects cells from FAS-mediated apoptosis through the cellular IL-10/PEA-15 pathway

**DOI:** 10.1099/vir.0.000180

**Published:** 2015-08

**Authors:** Emma Poole, Jonathan C. H. Lau, John Sinclair

**Affiliations:** University of Cambridge, Department of Medicine, Box 157, Level 5 Laboratories Block, Addenbrooke's Hospital, Hills Road, Cambridge CB2 0QQ, UK

## Abstract

Latent infection of primary CD34^+^ progenitor cells by human cytomegalovirus (HCMV) results in their increased survival in the face of pro-apoptotic signals. For instance, we have shown previously that primary myeloid cells are refractory to FAS-mediated killing and that cellular IL-10 (cIL-10) is an important survival factor for this effect. However, how cIL-10 mediates this protection is unclear. Here, we have shown that cIL-10 signalling leading to upregulation of the cellular factor PEA-15 mediates latency-associated protection of CD34^+^ progenitor cells from the extrinsic death pathway.

Human cytomegalovirus (HCMV), like all herpesviruses, has both a lytic and a latent phase to its infectious cycle. During the lytic life cycle, all of the viral genes are expressed in a temporal fashion. This results in major effects on the host cell, which include multiple strategies for host immune evasion (Amsler *et al.*, 2013; Jackson *et al.*, 2011; McSharry *et al.*, 2012; Noriega *et al.*, 2012; Weekes *et al.*, 2014). In contrast, during latent infection in, for example, cells of the myeloid lineage, there is a far more restricted transcription programme (Goodrum *et al.*, 2002; Rossetto *et al.*, 2013; Slobedman & Mocarski, 1999). Although the functions of these latency-associated gene products during latency have not yet been fully elucidated, it has been the focus of a number of studies (Avdic *et al.*, 2011; Keyes *et al.*, 2012; Mason *et al.*, 2013; Poole *et al.*, 2013; Reeves & Sinclair, 2010; Rossetto *et al.*, 2013; Weekes *et al.*, 2013), and it is now clear that latent infection of myeloid cells with HCMV can have profound effects on the latently infected cell.

For example, it has been shown that during latent infection there are a number of changes to the cellular microRNAome (Fu *et al.*, 2014; Poole, 2011), as well as changes in the cellular secretome (Mason *et al.*, 2012), and recently there have been a number of innate immune evasion functions found associated with short-term experimental HCMV latency in monocytes (Noriega *et al.*, 2014). The functions of some of the changed identified factors have been demonstrated (Mason *et al.*, 2012, 2013; Poole, 2011; Poole *et al.*, 2013) and at least one, IL-10, has significant pro-life effects on the latent cell (Mason *et al.*, 2012; Poole, 2011), including the inhibition of FAS-mediated apoptosis (Poole, 2011). The latency-induced upregulation of the pro-life factor IL-10 is mediated in part by downregulation of the microRNA hsa-miR-92a and upregulation of the anti-apoptotic factor Bcl2 (Poole, 2011). In addition to the upregulation of Bcl2, a full transcriptome analysis of monocytes latently infected with HCMV found changes in the levels of the related family member Mcl1 (Chan *et al.*, 2010). It is likely that this myeloid factor plays an important anti-apoptotic role in signalling mediated by virus binding to the cell surface, as well as during HCMV latency (Reeves *et al.*, 2012). Another study of the complete HCMV latent transcriptome in granulocyte–macrophage precursors also identified upregulation in mRNA levels of the anti-apoptotic factor PEA-15 (Slobedman & Mocarski, 1999), although changes in protein levels have not been confirmed. Interestingly, PEA-15 can be upregulated by IL-10 (Todaro *et al.*, 2006); however, whether there is a link between the observed upregulation of IL-10 and PEA-15 mRNAs during HCMV latency has not so far been demonstrated.

Therefore, we initially tested whether PEA-15 was upregulated at the protein level in primary CD34^+^ cells during HCMV latency. Fig. 1(a) shows that CD34^+^ cells infected with HCMV as described previously (Poole *et al.*, 2014b) and left to establish a latent infection contained, as expected (Poole *et al.*, 2014b), undetectable levels of IE72 RNA in the presence of detectable LUNA protein when tested by reverse transcriptase (RT)-PCR confirming that these cells had all the hallmarks of latent infection (Mason *et al.*, 2012; Reeves *et al.*, 2005). This was in contrast to lytic infection where IE72 RNA was detectable (Fig. 1a). Consequently, we also confirmed that the latency-associated increase in PEA-15 protein in CD34^+^ cells was also reflected at the level of mRNA (analysed using Qiagen RT-qPCR primers and a SYBR Green kit). Fig. 1(b) shows that quantitative RT-PCR analysis, as described previously (Poole, 2011), also showed upregulation of PEA-15 mRNA in latently infected CD34^+^ cells. Western blotting of cell lysate for PEA-15 (using an antibody from Cell Signalling) showed that, compared with the β-actin control (using an antibody from Abcam), there was a robust increase in the levels of PEA-15 protein following the establishment of latency in CD34^+^ myeloid progenitor cells. Our observations that PEA-15 is increased during latent infection of CD34^+^ progenitors at the level of the protein is consistent with a previous transcriptome analysis of latently infected granulocyte–macrophage progenitors (Slobedman & Mocarski, 1999).

**Fig. 1.**
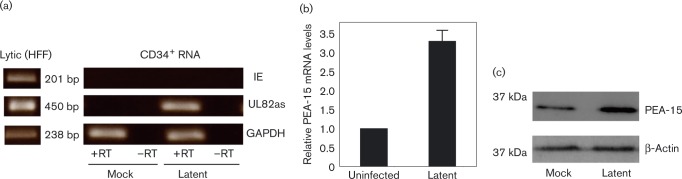
PEA-15 is upregulated during HCMV latency at the mRNA and protein levels. (a) Primary CD34^+^ myeloid progenitor cells were either uninfected (mock) or latency was established for 10 days and confirmed by reverse transcription (RT)-PCR analysis (right-hand panels). As a positive control, RT-PCR was also carried out on lytically infected fibroblasts (left-hand panel). (b, c) The cells were harvested for quantitative RT-PCR analysis for PEA-15 transcripts (b) or lysed for Western blot analysis for PEA-15 relative to a β-actin control (c). Data are representative of three replicates. Results in (b) are means ± sd. HFF, human foreskin fibroblasts; IE, immediate early; UL82as, UL82 anti-sense; GAPDH, Glyceraldehyde 3-phosphate dehydrogenase.

The data, so far, demonstrated that PEA-15 was upregulated during latency at both the mRNA and protein levels. However, in order to determine whether PEA-15 was directly involved in the ability of cellular IL-10 (cIL-10) to protect CD34^+^ cells from extrinsic death signals, it was necessary to assess the protection of CD34^+^ cells by cIL-10 in the absence of PEA-15. Unfortunately, primary CD34^+^ cells are notoriously difficult to manipulate by transfection, so we turned to the CD34^+^ cell line Kasumi-3, which is easily transfectable (Amaxa, Lonza; all transfections had an efficiency of >95 %, demonstrated with green fluorescent control) and reproduces many of the major aspects of latent HCMV infection. Importantly, although the Kasumi-3 myeloblastic cell lines appear to show some differences with respect to primary CD34^+^ cells in the establishment of latent infection with the AD169 laboratory isolate of HCMV, they fully support latent infection with clinical isolates of HCMV and are equivalent to primary CD34^+^ cells in this respect (Albright & Kalejta, 2013). Indeed, latent infection of Kasumi-3 cells results in all the hallmarks of latent infection observed in primary CD34^+^ cells, such as maintenance of the viral genome with concomitant lack of lytic IE72 expression but the presence of expression of the latency-associated LUNA gene (Albright & Kalejta, 2013; O'Connor & Murphy, 2012).

Initially, we confirmed that Kasumi-3 cells supported latent infection and reactivation in our hands by using a TB40E strain of HCMV, which expresses the lytic antigen IE2 fused to red fluorescent protein (RFP) (Fig. 2). Fig. 2 shows that after 3 days of infection there was no detectable IE2 protein (Fig. 2, latent), yet these cells were still able to reactivate virus after differentiation (Fig. 2, reactivation), again consistent with previous studies of IE72 protein expression in these cells (O'Connor & Murphy, 2012). As expected, cells that were differentiated prior to infection were permissive (Fig. 2, lytic). Therefore, we chose to use a 3-day time period for latent infection for subsequent small interfering RNA (siRNA) transfection analyses.

**Fig. 2.**
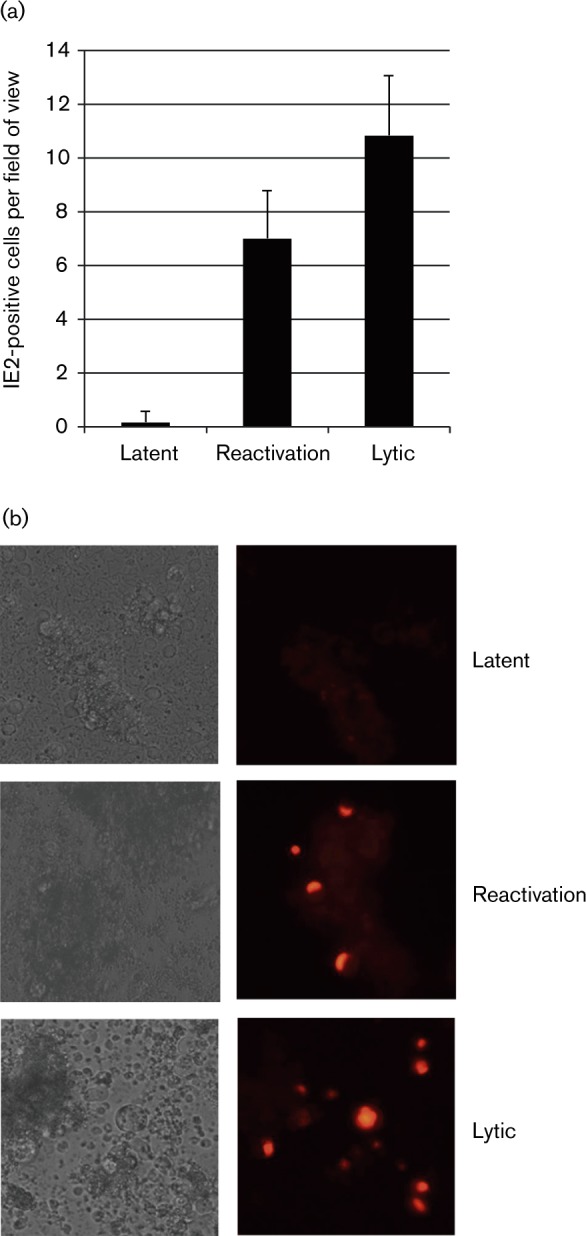
Latent infection is established in Kasumi-3 cells at 3 days post-infection. Undifferentiated Kasumi-3 cells were infected with IE2–RFP tagged HCMV TB40E strain at an m.o.i. of 3.0 for 3 days before being analysed (latent) or following the establishment of latency for 3 days. Kasumi-3 cells were treated with phorbol 12-myristate 13-acetate as described previously (O'Connor & Murphy, 2012) for 48 h before analysis (reactivation). Finally, prior to infection, cells were differentiated with phorbol 12-myristate 13-acetate for 48 h (lytic). Cells were counted and presented graphically (a) and were analysed directly by immunofluorescence (b). Data in (a) represent triplicate samples of six fields of view (means ± sd).

Having established that Kasumi-3 cells can be used as a model of latent infection and can also be manipulated by transfection, we tested the effect of removing PEA-15 on cell survival after extrinsic pro-death signals during latency by RNA interference knockdown. Consistent with previous findings for primary CD34^+^ cells (Poole, 2011), as expected, uninfected Kasumi-3 cells were clearly sensitive to FAS-mediated killing (Fig. 3a, lane 2), with minimal levels of apoptosis in the absence of FAS (Fig. 3a, lane 1). FAS-mediated killing was also inhibited by the pan-caspase inhibitor Z-VAD-FMK (Fig. 3A, lane 3), and control siRNA itself did not induce cell death in the presence of HCMV latent infection (Fig. 3a, lane 4). Also, consistent with previous analyses using primary CD34^+^ cells, latent infection of Kasumi-3 cells resulted in their protection from FAS-mediated killing (Fig. 3a, lane 5). To test whether PEA-15 was important for this resistance to FAS-mediated killing, we repeated the killing assay in cells in which we had knocked down PEA-15. Fig. 3(a, lane 8) shows that, in contrast to latently infected cells that had been treated with control siRNAs (Fig. 3a, lane 5, 22 % apoptosis), latently infected cells that had been treated with siRNAs to PEA-15 were sensitive to FAS-mediated cell killing (Fig. 3a, lane 8, 79 % apoptosis). Thus, the removal of PEA-15 from cells carrying latent HCMV prevented their resistance to FAS-mediated killing. These data argued that the latency-associated resistance of these CD34^+^ myelomonocytic cells to FAS-mediated killing was, at least in part, directly due to the presence of PEA-15. The effectiveness of PEA-15 removal from transfected latently infected cells using siRNAs was confirmed by Western blotting (Fig. 3b, anti-β-actin and anti-PEA-15 antibodies from Abcam); although PEA-15 was undetectable in untreated Kasumi-3 cells in our hands, when PEA-15 was induced by the establishment of HCMV latency, transfection with PEA-15-specific siRNAs in these cells eliminated PEA-15 protein expression (Fig. 3b).

**Fig. 3.**
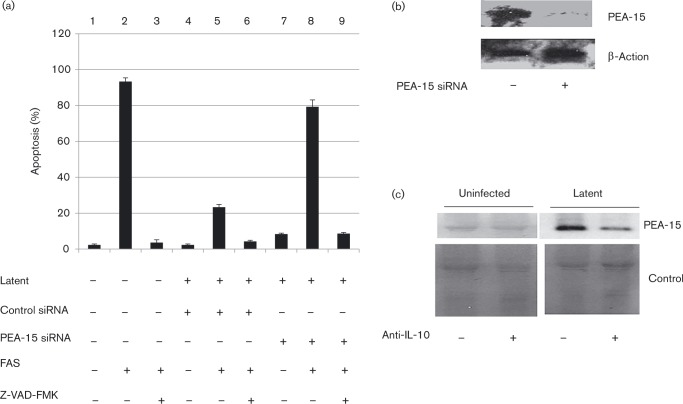
PEA-15 is involved in the evasion of FAS-mediated apoptosis and is induced by cIL-10. (a) Kasumi-3 cells were treated with control siRNA, siRNA to PEA-15 or untreated, and latency was then established for 3 days before treating with the apoptosis inducer FAS or the inhibitor of apoptosis Z-VAD-FMK as indicated and analysing for apoptosis, as described previously (Poole *et al.*, 2011). Data represent four replicates (means ± sd). (b) The latent cells from (a) that had been treated with siRNAs to PEA-15 were harvested for Western blot analysis and probed with anti-actin (Abcam) control primary antibody or anti-PEA-15 (Abcam) antibody. (c) Primary CD34^+^ cells were either uninfected (mock) or latency was established in the presence or absence of a neutralizing anti-IL-10 antibody before harvesting and blotting for PEA-15 against a Coomassie blue loading control.

Given that latency was not established in 100 % of the infected cell population, the very high percentages of cells resistant to FAS-mediated killing observed in the presence of PEA-15 during latency suggested that the mechanism by which PEA-15 confers protection is likely to be mediated via a secreted factor that would also protect uninfected bystander cells. It is known that the latency-induced secretome contains latency-induced cIL-10 (Mason *et al.*, 2012) and that this latency-associated increase in cIL-10 helps maintain latent viral genome carriage by preventing cell death (Poole, 2011; Weber-Nordt *et al.*, 1996); it also known that cIL-10 causes upregulation of PEA-15 (Todaro *et al.*, 2006). Consequently, we tested whether cIL-10 was required for the upregulation of PEA-15 during HCMV latency. To test this, a cIL-10 neutralization assay was carried out (antibodies from R&D Systems, neutralization carried out in accordance with the manufacturer's instructions). Fig. 3(c) shows that treatment of latently infected CD34^+^ cells with a neutralizing anti-cIL-10 antibody resulted specifically in substantially decreased levels of PEA-15 protein. Importantly, the cIL-10-neutralizing antibodies used were specific for cIL-10 and did not target the HCMV-encoded viral IL-10 homologue. Consequently, this argued that it is cIL-10, and not viral IL-10, that is the major factor required for the observed upregulation of the pro-life factor PEA-15.

PEA-15 is an anti-apoptotic molecule that potently regulates FAS/tumour necrosis factor receptor 1-induced apoptosis. This is achieved by targeting the Fas-associated death domain protein (FADD), an early effector of the death-inducing signalling complex (Todaro *et al.*, 2006). Consequently, latency-associated increases in PEA-15 would have profound effects on extrinsic pro-death signalling to the cell. Our observations that neutralizing antibodies to cIL-10 reduced the latency-associated increase in PEA-15 argue strongly that the increase in PEA-15 during latency was directly mediated by cIL-10. These data fit with the observation that cIL-10 causes upregulation of PEA-15 (Todaro *et al.*, 2006). Additionally, we have shown previously that cIL-10 from the latency-associated secretome helps latent cell survival (Poole, 2011; Poole *et al.*, 2014a; Mason *et al.*, 2012); removal of cIL-10 from latently infected CD34^+^ cells results in increased FAS-mediated apoptosis (Poole, 2011).

In conclusion, our data suggest that latent HCMV infection of CD34^+^ cells results in changes in the levels of anti-apoptotic cellular factor PEA-15, which are mediated via cIL-10 to enhance cell survival. We believe that this is further evidence that latent carriage of HCMV results in profound changes to the cell, which, without concomitant and coordinated changes in cell death signalling pathways, would lead to loss of the latently infected cell population.
